# Pathological Insights: Enhanced Vision Transformers for the Early Detection of Colorectal Cancer

**DOI:** 10.3390/cancers16071441

**Published:** 2024-04-08

**Authors:** Gelan Ayana, Hika Barki, Se-woon Choe

**Affiliations:** 1 Department of Medical IT Convergence Engineering, Kumoh National Institute of Technology, Gumi 39253, Republic of Korea; gelan@kumoh.ac.kr; 2 School of Biomedical Engineering, Jimma University, Jimma 378, Ethiopia; 3 Department of Artificial Intelligence Convergence, Pukyong National University, Busan 48513, Republic of Korea; daljuhika@pukyong.ac.kr; 4 Department of IT Convergence Engineering, Kumoh National Institute of Technology, Gumi 39253, Republic of Korea; 5 Emerging Pathogens Institute, University of Florida, Gainesville, FL 32608, USA

**Keywords:** vision transformer, spatial transformer, colorectal cancer, pathological findings, early detection, endoscopy

## Abstract

**Simple Summary:**

Accounting for 10% of the new cases in 2020, colorectal cancer (CRC) is one of the most prevalent cancers worldwide. Unfortunately, CRC is frequently identified at a later stage, despite the fact that early detection greatly increases survival rates. Diagnostic endoscopy is the gold standard; however, identifying abnormalities at an early stage is challenging. In particular, convolutional neural networks (CNNs) are being used by researchers to improve detection through deep learning. But prior approaches were primarily concerned with polyp detection. This work provided a novel method for polyp segmentation and endoscopic pathological finding categorization using vision transformers and spatial transformers for the early identification of colorectal cancer (CRC). These approaches perform noticeably better than the current CNN-based algorithms. This work opens up exciting possibilities for improving on early CRC detection beyond just identifying polyps.

**Abstract:**

Endoscopic pathological findings of the gastrointestinal tract are crucial for the early diagnosis of colorectal cancer (CRC). Previous deep learning works, aimed at improving CRC detection performance and reducing subjective analysis errors, are limited to polyp segmentation. Pathological findings were not considered and only convolutional neural networks (CNNs), which are not able to handle global image feature information, were utilized. This work introduces a novel vision transformer (ViT)-based approach for early CRC detection. The core components of the proposed approach are ViTCol, a boosted vision transformer for classifying endoscopic pathological findings, and PUTS, a vision transformer-based model for polyp segmentation. Results demonstrate the superiority of this vision transformer-based CRC detection method over existing CNN and vision transformer models. ViTCol exhibited an outstanding performance in classifying pathological findings, with an area under the receiver operating curve (AUC) value of 0.9999 ± 0.001 on the Kvasir dataset. PUTS provided outstanding results in segmenting polyp images, with mean intersection over union (mIoU) of 0.8673 and 0.9092 on the Kvasir-SEG and CVC-Clinic datasets, respectively. This work underscores the value of spatial transformers in localizing input images, which can seamlessly integrate into the main vision transformer network, enhancing the automated identification of critical image features for early CRC detection.

## 1. Introduction

Colorectal cancer (CRC), excluding skin cancer, is the third most common cancer by incidence and the second most common cause of death among other cancers in both sexes [1]. For several decades, the death rate from colorectal cancer in both sexes has decreased [2]. One of the reasons is that colorectal polyps are now more frequently discovered through screening and removed before they can progress to cancer, or tumors are discovered sooner when they can be treated more effectively [3,4]. Additionally, colorectal cancer treatments have advanced, resulting in more than 1.5 million colorectal cancer survivors in the United States alone [1,5]. Despite the fact that the total mortality rate has continued to decline, colorectal cancer mortality among those younger than 55 years has increased by 1% annually since 2008 [5,6]. One of the most effective ways to prevent colorectal cancer is routine screening [7]. When colorectal cancer is in its early stage, it is frequently smaller, has not spread, and may be easier to treat. It can take up to 10 or 15 years for a polyp to become cancerous [8,9]. Doctors can detect and remove polyps through screening before they become cancerous. As CRC that is identified at an early stage has a five-year relative survival rate above 90%, early detection of the disease is essential [10]. However, only 40% of CRC cases are found in the early stages, and the survival probability drops if the disease spreads outside the colon or rectum [11]. Endoscopy is recommended for the diagnosis of CRC when clinicians notice an abnormal growth in the colon or rectum [10]. Endoscopic observation of CRC mostly focuses on finding polyps; however, esophagitis and ulcerative colitis might also be related to CRC [12,13]. Studies have found that esophagitis and CRC share risk factors, and that the rate of CRC is significantly increased in the presence of esophagitis [12,14,15,16]. Furthermore, patients with ulcerative colitis are at a higher risk of developing colorectal cancer [17,18]. This phenomena of esophagitis and ulcerative colitis cases increasing the risk of CRC affects the early diagnosis of CRC [19,20,21]. This warrants a mechanism for the early identification of esophagitis, ulcerative colitis, and polyps, so that efficient CRC treatment can be administered. Esophagitis, ulcerative colitis, and polyps are referred to as pathological findings under endoscopy [8,22,23]. Early identification of gastrointestinal endoscopic pathological findings is crucial for the early detection and treatment of CRC [5,8,10]. However, the detection and identification of endoscopic pathological findings at an early stage are cumbersome [24,25]. Deep learning has been sought by researchers to overcome the general challenges of early detection of endoscopic CRC, and it has brought about significant improvements, especially in polyp detection [26,27]. Although extensive research has been conducted on polyp detection, studies involving esophagitis and ulcerative colitis for the early diagnosis of CRC are not available. Moreover, most state-of-the-art deep learning studies for early detection of CRC are solely based on convolutional neural networks (CNNs) [28,29,30]. CNNs can learn visual representations for easy transfer and strong performance, owing to the strong inductive bias of spatial equivariance and translational invariance provided by their convolutional layers [31,32,33,34,35]. However, vision transformers (ViTs) exhibit superior performance over CNNs for natural image classification and segmentation [36,37,38,39]. In contrast to CNNs, which perform many convolutions at different layers to focus on a given area of an image, ViT focuses on all areas of the image at the beginning of its early layers [40,41]. Despite the numerous applications of CNNs to endoscopic colorectal images, ViT has not yet been explored for its use in the detection of CRC [42,43,44]. In this study, we propose a novel deep learning algorithm for the classification of endoscopic pathological findings and polyp segmentation based on ViTs for the early detection of CRC. The contributions of this study are as follows:

- Inclusion of other pathological findings in addition to polyp for early CRC diagnosis;
- ViTCol: Boosted ViT endoscopic pathological findings classifier for early CRC detection;
- PUTS: A novel Swin-Unet transformer-based polyp segmentation model;
- Comparison of ViTCol and PUTS with state-of-the-art CNN and ViT methods.

This study outlines several novel contributions to the field of CRC detection. Firstly, it breaks away from the conventional approach that is solely focused on polyp detection by incorporating additional pathological findings, such as esophagitis and ulcerative colitis, into the early diagnosis of CRC. This expanded scope enhances the comprehensiveness of CRC detection methodologies. Secondly, the introduction of ViTs represents a novel departure from the predominant use of CNNs in CRC detection. ViTs offer superior performance in image classification and segmentation tasks, potentially revolutionizing the accuracy and efficiency of CRC detection. Thirdly, the proposed deep learning models, ViTCol for endoscopic pathological findings classification and PUTS for polyp segmentation, introduce innovative solutions tailored specifically for CRC detection tasks. These models present advancements in both classification and segmentation aspects crucial for early CRC diagnosis. Lastly, the comparative analysis conducted against state-of-the-art CNN and ViT methods highlights the unique strengths and potential superiority of the newly proposed models, further emphasizing their novelty and contribution to advancing CRC detection methodologies. Overall, this study introduces a multifaceted approach to CRC detection, encompassing expanded pathological findings, innovative deep learning models, and comparative evaluation, thereby significantly advancing the state-of-the-art methods of CRC detection.

## 2. Related Works

A few studies have applied ViTs to the detection of colorectal cancer [39,45,46,47,48,49,50]. All these studies focused only on polyp images, and the target task was the semantic segmentation of these polyps. The main objective of previous studies was to compensate for the global semantic information loss by CNNs without sacrificing the low-level and local information from CNNs. Park et al. [39] combined CNN and ViT for polyp segmentation to design a robust model for maintaining global semantics without sacrificing the low-level features of CNNs. The authors combined EfficientNet (CNN) and Swin transformer (ViT) to achieve their objectives and reported improved performance compared to current state-of-the-art methods. Li et al. [45] also proposed a model that combined CNN and ViT, a multi-information aggregation network (MIA-Net) for polyp segmentation, to overcome the intra- and inter-class inconsistencies in previous approaches. Using a powerful global contextual feature extraction module, they used a ViT to extract global characteristics and pinpoint polyps more accurately. The impact of intraclass inconsistency was lessened by this strategy. In addition, their model aggregated the high- and low-level data recovered by the transformer with fine-grained local texture features captured by the convolutional encoder. The model became more sensitive to edge information, and it reduced interclass indistinctions owing to this rich feature information. According to their experimental findings, the MIA-Net exhibited excellent generalization and good fitting capabilities. In addition, MIA-Net surpassed previous algorithms and considerably increased the accuracy of polyp segmentation. Pan et al. [46] designed a fusion of a transformer and a CNN for polyp segmentation called EG-TransUNet. A progressive enhancement module (PEM) and channel spatial attention (CSA) were used to create the EG-TransUNet architecture, a U-Net variant that can increase feature discrimination at the level of spatial detail and semantic placement. Based on self-attention, CSA uses semantic guidance attention (SGA) to seamlessly combine spatial and semantic information. The authors claimed that EG-TransUNet could capture object variability and provide better results on various biological datasets, including polyp images. The CNN backbone used was ResNet50. Similarly, Duc et al. [47] designed a combined transformer and CNN network called ColonFormer, which was motivated by earlier methods for modeling multi-scale and multi-level characteristics. The transformer was used as an encoder, and a CNN was used as a decoder in the ColonFormer. The encoder in ColonFormer is a lightweight, hierarchically organized transformer that can learn multi-scale characteristics. The decoder employs a hierarchical pyramid structure and can learn from heterogeneous inputs that include feature maps taken from encoder blocks at various scales and subregions. Additionally, a refinement module was included to further boost the segmentation accuracy on difficult locations and tiny polyps. ColonFormer outperformed the previous methods in polyp segmentation. Other studies that focused on improving polyp segmentation by detecting challenging features [49] and compensating for feature fusion from ViTs and CNNs [48] have also been proposed and shown to improve polyp segmentation.

However, most of the previous studies focused only on polyp segmentation, not considering other pathological findings for colorectal cancer detection and the combination of ViTs and CNNs as encoders or decoders. Little attention was given to preprocessing the input images so that all the necessary features were extracted accurately for ViTs to be able to capture all local and global information simultaneously.

The current work improves upon the limitations of previous studies in several ways. Firstly, while previous studies solely focused on polyp segmentation using ViTs and CNNs, the current study expands the scope by considering other pathological findings crucial for colorectal cancer detection. By incorporating additional pathological findings beyond polyps, the current work enhances the comprehensiveness of CRC detection methodologies, addressing a notable gap in existing research. Secondly, previous studies utilized ViTs and CNNs primarily as either encoders or decoders in isolation. In contrast, the current study proposes a novel approach by combining ViTs and CNNs as both encoders and decoders, leveraging the strengths of both architectures to extract and process features effectively. This integrated approach ensures comprehensive feature extraction and processing, improving the accuracy and robustness of CRC detection models. Additionally, the current study addresses the issue of feature extraction accuracy by emphasizing the importance of preprocessing input images to accurately capture all necessary features for ViTs to effectively capture both local and global information simultaneously. By focusing on preprocessing techniques to optimize feature extraction, the current work ensures that ViTs can leverage the full spectrum of information available in the input data, leading to more accurate and reliable CRC detection outcomes. Overall, by expanding the scope of the pathological findings considered, integrating ViTs and CNNs in a novel manner, and emphasizing preprocessing techniques for enhanced feature extraction, the current study significantly advances CRC detection methodologies beyond the limitations of previous research efforts.

## 3. Materials and Methods

We developed a novel deep learning algorithm for the classification of endoscopic pathological findings and polyp segmentation based on a ViT network for the early diagnosis of CRC, as described below.

### 3.1. ViTCol: Vision Transformer-Based Classifier for CRC Detection

#### 3.1.1. Motivation 

Vision transformers enable a global view of an image from the first layer without losing data regarding the position and orientation of an object. These advantages of ViTs become more significant when smaller patch sizes are used or when the input images are smaller. To achieve a smaller input image size while focusing on the important parts of the image for CRC detection, we developed a boosted ViT model with a spatial transformer module for weakly localizing the input image. 

#### 3.1.2. ViTCol Architecture

The proposed architecture has a spatial transformer network (STN) as a module that helps localize the important part of an input image and feeds its output into the ViT architecture for attention. The spatial transformer generates a localization of the input image as its output, which is converted into patches to be fed into the transformer encoder network [51]. 

**Figure 1:**
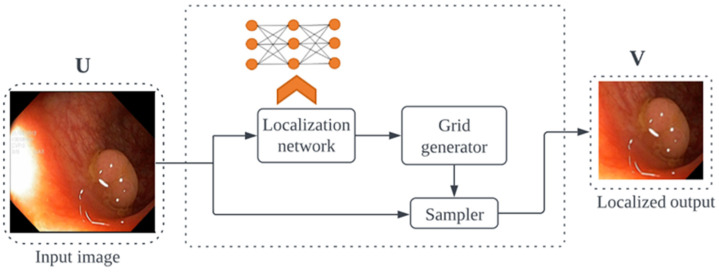
Localization module. U: input image, and V: localized output image.

The localization module has three parts: localization network, grid generator, and sampler (Figure 1). The localization network generates θ parameters, which are learned as affine transforms for input U with width W, height H, and channel C. The affine transformation is given by (1).

(1) $$\left(\begin{array}{c} x_{i}^{s}\\ y_{i}^{s} \end{array}\right)=T_{\theta }\left(G_{i}\right)=A_{\theta }\left(\begin{array}{c} x_{i}^{t}\\ y_{i}^{t}\\ 1 \end{array}\right)=\left[\begin{array}{ccc} \theta_{11} & \theta_{12} & \theta_{13}\\ \theta_{21} & \theta_{22} & \theta_{23} \end{array}\right]\;\left(\begin{array}{c} x_{i}^{t}\\ y_{i}^{t}\\ 1 \end{array}\right)$$

The grid generator calculates the relevant sample positions in the input image using $T_{\theta }$ on G  transformation as it iterates across the target image’s regular grid. The result is a collection of points with target coordinates ($x_{i}^{t}$, $y_{i}^{t}$), which were modified based on the transformation parameters. The sampler creates an output feature map V based on a new set of coordinates ($x_{i}^{t}$, $y_{i}^{t}$).

The output of the localization module was fed into a ViT model. A transformer model is a type of neural network that tracks relationships in sequential data, similar to words in a phrase, to learn meaning and context. Data sequences in the form of image patches are used by vision transformers. The ViT operates as follows.

**Figure 2:**
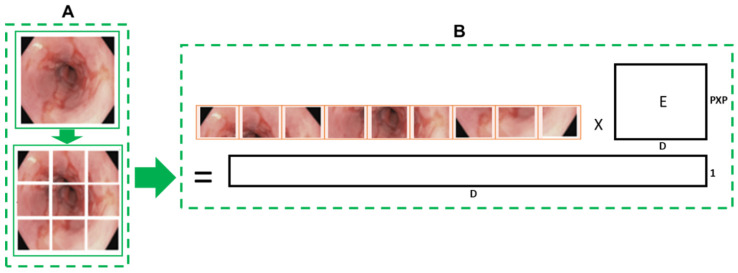
Vision transformer image sequence formation. (**A**) Image patch formation. (**B**) Flattening and patch embedding. *P* × *P*: patch size, D: latent vector size for linear projection, and E: randomly generated matrix.

**Figure 3:**
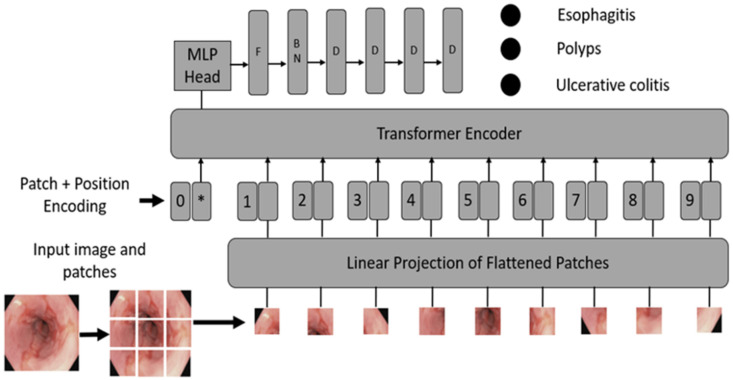
The proposed ViTCol architecture. * extra learnable (class) embedding, 1,2,…,9: index of patches, *MLP*: multilayer perceptron, F: flatten, BN: batch normalization, and D: dense layer.

- Convert images into patches: Image patches are used as tokens of words by vision transformers, as in the original paper by Vaswani et al. [52]. For images, it is possible to take pixels; however, if pixels are considered, the computational cost will be high, and it is challenging to find hardware to process high-resolution images, as in the case of medical images. Thus, converting input images into patches has been proposed by Dosovitskiy et al. [36], as shown in Figure 2A. An image with H×W is converted into N patches of size P×P.
- Flattening and patch embedding the patches: After that, a single feed-forward layer is applied to the flattened patches to create a linear patch projection, as shown in Figure 2B. This feedforward layer contains the embedding matrix E as mentioned by [36]. Matrix E was randomly generated.
- Learnable and positional embeddings: Patch projection is concatenated together with learnable embeddings, which are used later for classification. Transformers use positional embeddings to construct a certain order in the patches since, unlike time sequence models, patches do not spontaneously form into sequences. Similar to patch embedding, the positional encoding matrix is produced at random.
- Multilayer perceptron (*MLP*) head: The *MLP* head receives the transformer encoder unit’s outputs for classification. Despite the multiple outputs of the transformer encoder, the *MLP* takes only one output related to the class embedding, while the other outputs are ignored. The probability distribution of the labels that the associated images belong to is output by *MLP*. Figure 3 shows the ViT architecture used in ViTCol.

#### 3.1.3. Model Definition

The goal is to learn a mapping ∅, between two variables $x_{P}$ and t,$\emptyset :\;x_{P}{\in X}^{N\times (P\times P\times C)}\;\to \;t\in T$, where $x_{P}$ stands for the D dimensional patches and T stands for the output class space. In other words, we search for a mapping between the output class probabilities for each series of input picture patches. A transformer encoder unit and *MLP* head were used to implement this mapping. With its two components, an encoder and decoder, the transformer encoder unit converts the input patch sequence into an output. Multi-head attention and feed-forward layers constitute the majority of the encoder and decoder components.

The attention mechanism is given by Equation (2).

(2) $$A\left(Q,K,V\right)=softmax(\frac{{QK}^{T}}{\sqrt{d_{k}}})V$$

where Q is the vector representation of a single patch from the input sequence, K is the vector representation of all patches as keys, and V is the vector representation of all patches as values. SoftMax produces values in the range of 0–1. The feedforward algorithm then continues after the multi-head attention processes in the encoder and decoder. Using feedforward layers, a distinct linear transformation is performed for each component of the sequence. The features are then transformed into an output classification function by the *MLP* using the transformer encoder outputs. The transformer encoder offers several outputs, but the *MLP* chooses only one output that closely resembles the class being considered and disregards the other outputs. The probability distribution of the corresponding classes under which the image is classified is output by *MLP*.

The pathological finding classes were encoded as probability distributions using the SoftMax output. As a result, when it comes to training the network, we simply use cross-entropy, as shown in Equation (3), where C is the collection of potential classes for the images.

(3) $$Loss=-\sum_{i=0}^{\left|C\right|}{\hat{c}}_{i}\log\left(\frac{exp\left(\emptyset {\left(X\right)}_{i}\right)}{\sum_{j}^{\left|C\right|}exp\left(\emptyset {\left(X\right)}_{j}\right)}\right)$$

#### 3.1.4. Implementation

**Figure 4:**
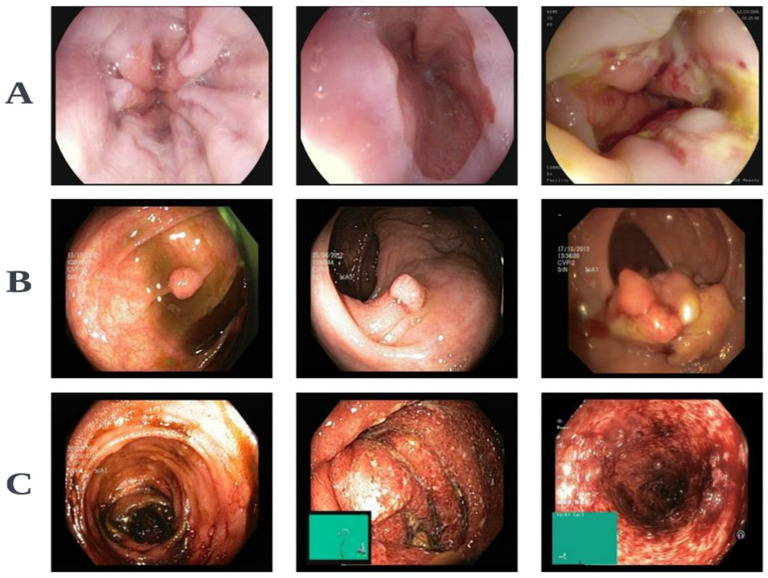
Sample images from the Kvasir dataset (**A**) Esophagitis. (**B**) Polyps. (**C**) Ulcerative colitis.

The ViTCol method utilized three different ViT base models: vitb_16, vitb_32, and vitl_32. An Adam optimizer with a learning rate of 0.0001 was used. The proposed model was run for 50 epochs, with a batch size of 16. We used L2 regularization and the Gaussian error linear unit (GELU) activation function. Augmentation was used to enrich the training dataset. Several augmentations were used, including random rotation (range = 40), flip (horizontal and vertical, range = 0.2), shift (width and height, range = 0.2), shear (0.1), and zoom (0.1). We used a publicly available gastrointestinal endoscopic pathological findings dataset called Kvasir (available online at https://datasets.simula.no/kvasir/ (last accessed on 5 April 2024). Sample images from this dataset are shown in Figure 4. Pathological findings were categorized into three classes: esophagitis, polyps, and ulcerative colitis, with 1000 images in each class. The pathological findings dataset was categorized into training and testing using a 9:1 ratio. The input image size was 224×224 and the patch sizes were 16×16 and 32×32, based on the base model type used. The model parameters were initialized using weights that were pretrained on ImageNet. Experiments were run on an RTX 3090 GPUs in Python 3.6, using the TensorFlow framework.

### 3.2. PUTS: Vision Transformer-Based Polyp Segmentation

#### 3.2.1. Motivation

U-Net-based models are popular for polyp segmentation. In general, these models focus on updating the encoder, decoder, skip connection, progressive supervision, data augmentation, and loss function to enhance the classical U-net network for improved polyp segmentation. These approaches have weaknesses in handling challenging polyp segmentation tasks, such as shape or size disparity, background noise, and vague boundaries. Moreover, existing studies are mostly based on CNNs, which create image features using convolutional kernels. Each convolutional kernel can only focus on one subregion of the entire image because of the inherent inductive biases, which cause it to lose the global context and fail to develop long-range dependencies. The downsampling process and convolution layer stacking increase the receptive field and improve the local interaction; however, this is not the best option because it complicates the model and makes it more susceptible to overfitting. To model long-range dependencies, self-attention mechanisms using transformers have been proposed and have improved medical image segmentation. However, transformers are computationally complex and require more resources than CNNs do. To reduce computation costs, window-based multi-head self-attention and shifting window-based multi-head self-attention hierarchical Swin transformers are considered. This leads to the creation of a Swin-Unet segmentation model that improves the performance of medical image segmentation. Nevertheless, the loss of shallow characteristics, such as edges and line information, will result from this method’s disregard for the intrinsic structural features at the pixel level within each patch. Therefore, we designed a novel Swin-Unet transformer-based polyp segmentation model (PUTS) to address the limitations of the current study. On one hand, we introduced a localization module to improve the performance of challenging polyp segmentation tasks. On the other hand, we used the Swin-Unet transformer with spatial transformer as our encoder and decoder to improve the Swin-Unet performance in feature extraction, overcoming the limitations of intrinsic structure feature extraction.

#### 3.2.2. PUTS Architecture

**Figure 5:**
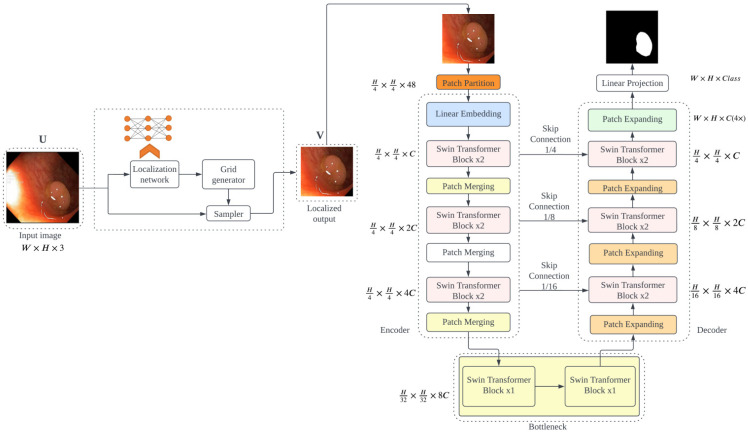
The architecture of PUTS, which is composed of a localization module and Swin-Unet architecture. U: input image, V: localized output image, H: height, W: width, and C: channel.

The proposed architecture has a spatial transformer module that helps to localize an important part of an input image and feed its output into the Swin-Unet architecture with a Swin transformer encoder and decoder. The spatial transformer generates a localization of the input image as its output, which is converted into patches to be fed into the Swin transformer encoder network, as in ViTCol. The Swin transformer extracts global contexts using tokenized patches from an input image feature map as the input sequence. Then, to enable accurate localization, the decoder samples the encoded features before combining them with high-resolution feature maps. The Swin transformer encoder harvests features at different resolutions using shifting windows for computing self-attention. At each resolution, skip connections connect it to a Swin transformer-based decoder, as in [38]. The proposed method is illustrated in Figure 5.

#### 3.2.3. Model Definition

Let $X=R^{i\times j}$ and Y be the input image and output segmentation mask spaces, respectively. For the segmentation task, we must distinguish between the polyp and non-polyp parts, denoted $S=\left\{0,\;1\right\}$. As such, $Y={\left\{0,\;1\right\}}^{i\times j}$. Our goal is to learn mapping Φ :X→Y, which provides an input endoscopic image and outputs the corresponding polyp segmentation mask. To design Φ, we propose a PUTS that has a localization module based on a spatial transformer and an encoder and decoder based on the Swin-Unet architecture.

The localization module produces an affine transformation of the input image as in the ViTCol architecture. It generates θ parameters, which are learned as affine transforms for input U with width W, height H, and channel C. The grid generator calculates the relevant sample positions in the input image using $T_{\theta }$ on G  transformation as it iterates across the target image’s regular grid. The result is a collection of points with target coordinates ($x_{i}^{t}$, $y_{i}^{t}$), which were modified based on the transformation parameters. Following this, the sampler creates an output feature map V based on the new set of coordinates ($x_{i}^{t}$, $y_{i}^{t}$), as shown in Equation (1).

**Figure 6:**
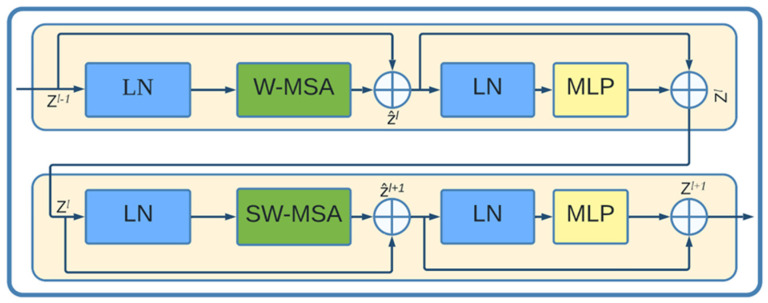
The Swin transformer block structure. Z stands for output features, *W-MSA* for window-based multi-head self-attention, *SW-MSA* for shifted window-based multi-head self-attention, *LN* for layer normalization, and *MLP* for multilayer perceptron.

The encoder, bottleneck, decoder, and skip connections make up Swin-Unet. A Swin Transformer Block is the fundamental building block of Swin-Unet. A Swin transformer block is built using shifted windows, in contrast to the traditional multi-head self-attention (*MSA*) module. Two sequential Swin transformer blocks are shown in Figure 6. Each Swin transformer block consists of a residual connection, multi-head self-attention module, layer normalization (*LN*) layer, and two-layer *MLP* along GELU nonlinearity. The two subsequent transformer blocks each employ window-based multi-head self-attention (*W-MSA*) and shifted window-based multi-head self-attention (*SW-MSA*) modules. Continuous Swin transformer blocks based on such a window partitioning approach can be given as in (7).

(4) $${\hat{z}}^{l}=W-MSA\left(LN\left(z^{l-1}\right)\right)+z^{l-1}$$

(5) $$z^{l}=MLP\left(LN\right)\left({\hat{z}}^{l}\right)+{\hat{z}}^{l}$$

(6) $${\hat{z}}^{l+1}=SW-MSA\left(LN\left(z^{l}\right)\right)+z^{l}$$

(7) $$z^{l+1}=MLP\left(LN\left({\hat{z}}^{l+1}\right)\right)+{\hat{z}}^{l+1}$$

In the Equations (4) and (5), ${\hat{z}}^{l}$ and $z^{l}$ represent the outputs of the *SW-MSA* module and the $l^{th}$ block *MLP* module, respectively. Self-attention is given as in (8).

(8) $$A\left(Q,K,V\right)=SoftMax(\frac{{QK}^{T}}{\sqrt{d_{k}}}+B)V$$

Q is the vector representation of a single patch from the input sequence, K is the vector representation of all patches as keys, and V is the vector representation of all patches as values. B is the bias and d is the dimension of the key.

In the encoder, the images that are generated by the localization module are split into patch sizes of 4×4, which are nonoverlapping, as in [38]. With C channels, the dimension of the feature of every patch is 4×4×C. The patches are then linearly embedded to obtain a linear patch feature projection. To create hierarchical feature representations, the changed patch tokens are passed through a number of Swin transformer blocks and patch merging layers. The downsampling and increasing dimensions are handled by the patch merging layer, whereas feature representation learning is handled by the Swin transformer block. In the decoder, we have a Swin transformer block and patch expanding layer, as in [38]. To compensate for the loss of spatial information brought on by downsampling, the recovered context features are fused with multi-scale data from the encoder via skip connections. The patch-expanding layer performs the upsampling task. With a two-time upsampling of resolution, the patch expanding layer reshapes the feature maps of the adjacent dimensions into a large feature map. After performing upsampling four times to obtain the resolution of the feature maps back to the input resolution (H×W), the last patch expanding layer is used to apply a linear projection layer on the upsampled features, which then produces the predictions for pixel-level segmentation.

#### 3.2.4. Implementation

**Figure 7:**
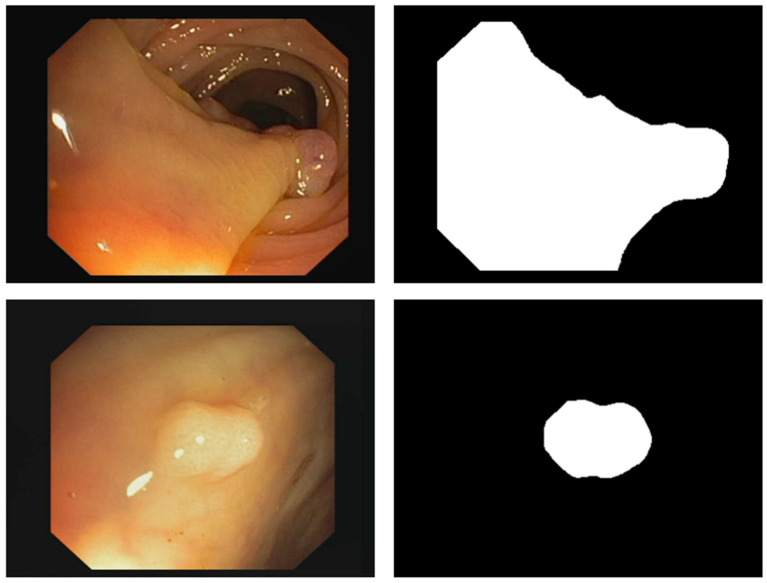
Examples of polyp pictures from the Kvasir-SEG dataset together with the masks that go with them.

Two publicly available polyp image datasets, Kvasir-SEG (available online at https://datasets.simula.no/kvasir-seg/ (last accessed on 5 April 2024)) and CVC-ClinicDB (available online at https://www.kaggle.com/datasets/balraj98/cvcclinicdb (last accessed on 5 April 2024)), were used to evaluate PUTS. Kvasir-SEG contains 1000 polyp images, and the CVC-ClinicDB dataset contains 612 polyp images. Sample images of polyps and their corresponding masks are shown in Figure 7. We split our dataset into 80% training and 20% testing sets. Given N image–mask pairs during training $T={\left\{\left(x_{n},\;y_{n}\right)\right\}}_{n=1}^{N}$, where $x_{n}\;\epsilon \;X$ and $y_{n}\;\epsilon \;Y$, the model parameters are learned via back-propagation of categorical cross-entropy using an Adam optimizer with a learning rate of 0.0001. In this study, we employed a pixel-wise categorical cross-entropy loss between the segmentation masks $g(y_{n})$ and model predictions ${\hat{y}}_{n}=\Phi (h(x_{n}))$. Every image–label pair is subjected to extensive online manipulation during training via a set of augmentation functions that were each triggered on the image–label pair to promote invariance to common polyp image transformations and hence, enhance the generalization of inference. We included augmentations such as random rotation (range = 40), flip (horizontal and vertical, range = 0.2), shift (width and height, range = 0.2), shear (0.1), and zoom (0.1). Training was stopped after 150 epochs. The input image size was 224×224 and the patch size was 4×4. The model parameters were initialized using weights that were pretrained on ImageNet. Experiments were run on an RTX 3090 GPUs in Python 3.6, using the TensorFlow framework.

### 3.3. Parameters Selection

Based on recent studies [53,54,55] on deep learning for CRC, we selected the following five optimizers: Adam, Adadelta, Adamax, Adagrad, and stochastic gradient descent (SGD). Next, we chose an activation function and ran tests using three different activation functions as follows: rectified linear unit (ReLu), exponential linear unit (ELU), and GELU. Based on the results, we selected GELU. Furthermore, we used three regularization techniques, L1, L2, and L1/L2, and selected L2 regularization based on performance. Furthermore, we thoroughly investigated the spectrum of learning rates to establish the ideal value for each optimizer, as the learning rate value had a significant impact on performance. We performed a grid search to find the best learning rate for each optimizer, which included the following five learning rate values: 1, 0.1, 0.01, 0.001, and 0.0001 with early stop. Based on the data, we used a learning rate of 0.0001 with the Adam optimizer to achieve the best outcome. Following the selection of the ideal learning rates, we conducted an experiment to determine whether to employ fixed epochs or early halting during training rounds. We employed 50, 100, 150, 200, 250, and 300 fixed epochs, and 300 early stopping tests with patience of 5, 7, and 10. Based on the experimental performance results and computational time, we chose a fixed epoch of 50 for ViTCol and 150 epochs for PUTS.

## 4. Results

### 4.1. Pathological Findings Classification Performance Results

To evaluate the performance of our proposed ViTCol CRC early detection method and its constituent parts, we considered the following evaluations: (i) ViTCol method using three ViT base models (vitb_16, vitb_32, and vitl_32); (ii) conventional ViT method using three base models (vitb_16, vitb_32, and vitl_32) without the localization module as in ViTCol; and (iii) comparison using three state-of-the-art CNN models (ResNet50, EfficientNetB2, and InceptionV3). We evaluated the pathological finding classification performance experiments carried out in this study in terms of accuracy, AUC, sensitivity, specificity, and F1-score. Because these metrics are prone to misdiagnosis and cannot provide exact performance, we also report the results generated using two additional metrics: Matthew’s correlation coefficient (MCC) and Cohen’s kappa values.

As shown in Table 1, ViTCol with the vitb_16 baseline performed best among the three ViT baselines in classifying pathological findings with an accuracy, AUC, sensitivity, specificity, F1-score, MCC value, and Kappa value of 0.9998 ± 0.001, 0.9999 ± 0.001, 0.9998 ± 0.001, 0.9998 ± 0.002, 0.9999 ± 0.001, 0.9995 ± 0.002, and 0.9997 ± 0.001, respectively. The vitb_32 and vitl_32 base models also provided comparable performance to the vitb_16 model. This demonstrated the effectiveness of the proposed ViTCol approach for classifying endoscopic pathological findings for early CRC detection. In terms of computational complexity, it took approximately 40 min to train ViTCol with the vitb_16 base model.

To determine the effectiveness of the additional constituent parts of ViTCol in improving the performance, we compared it with conventional ViTs using the same base models but without adding the localization module as in the ViTCol method. Consequently, as shown in Table 2, the vitb_16 base model provided the highest performance values of 0.9845 ± 0.001, 0.987 ± 0.001, 0.9832 ± 0.002, 0.9836 ± 0.003, 0.984 ± 0.001, 0.9817 ± 0.002, and 0.9826 ± 0.002 for accuracy, AUC, sensitivity, specificity, F1-score, MCC value, and kappa value, respectively. Compared to the ViTCol method, the conventional ViT method performed lower for all three base models used: vitb_16 (AUC of 0.987 ± 0.001 for conventional ViT method vs. 0.9999 ± 0.001 for ViTCol method), vitb_32 (AUC of 0.9742 ± 0.001 for conventional ViT method vs. 0.9882 ± 0.001 for ViTCol method), and vitl_32 (AUC of 0.969 ± 0.001 for conventional ViT method vs. 0.9774 ± 0.002 for ViTCol method). This demonstrated the superiority of the proposed ViTCol method over the conventional ViT method for classifying endoscopic pathological findings for early CRC detection.

Table 3 depicts the results generated for classifying endoscopic pathological findings using the following state-of-the-art CNN methods: ResNet50, EfficientnetB2, and InceptionV3. ResNet50 performed slightly better than EfficientnetB2 and InceptionV3, with an AUC of 0.9749 ± 0.001, 0.9707 ± 0.004, and 0.9718 ± 0.001 for ResNet50, EfficientnetB2, and InceptionV3, respectively. From the results in Table 1 and Table 3, it can be observed that the proposed ViTCol method outperforms the CNN method. Even with the worst performing base model (vitl_32), ViTCol outperformed the best performing model (ResNet50) of the CNN approach (AUC of 0.9774 ± 0.002 for ViTCol with vitl_32 base model vs. AUC of 0.9749 ± 0.001 for ResNet50 based CNN method). ViTCol models outperform CNNs due to their superior ability to capture long-range dependencies and global context in images. Unlike CNNs, which process images in a hierarchical manner through local receptive fields, ViTCol models process the entire image at once using self-attention mechanisms, allowing them to efficiently capture both local and global features. This enables ViTCol models to learn more robust representations of complex patterns and relationships within images, leading to improved performance.

### 4.2. Polyp Segmentation Performance Results

A quantitative assessment and comparison of segmentation performance for U-net, ResUnet, Unet3+, Deeplabv3, Trans-Unet, Swin-Unet, and the proposed PUTS models are provided in Table 4. Evaluation metrics include mean intersection-over-union (mIoU), mean Dice (mDic) score, sensitivity, specificity, and MCC. We observed that using the localization module in combination with the Swin-Unet model in PUTS yields a strong performance gain over the conventional Swin-Unet, increasing the mIoU, mDic, sensitivity, specificity, and MCC values from 0.8206, 0.8892, 0.8734, 0.8783, and 0.8642, respectively, to 0.8673, 0.9186, 0.9095, 0.9104, and 0.9048, respectively, for segmenting the Kvasir-SEG polyp image dataset. Similarly, on the CVC-Clinic dataset, PUTS using Swin-Unet performed better than the conventional Swin-Unet model, increasing mIoU, mDic, sensitivity, specificity, and MCC value, from 0.8552, 0.9053, 0.8937, 0.8915, and 0.8906, respectively, to 0.9092, 0.9484, 0.9392, 0.9387, and 0.9317, respectively.

Furthermore, the proposed PUTS polyp segmentation method outperformed the state-of-the-art segmentation models on the Kvasir-SEG dataset irrespective of whether the models were purely CNN, as in the case of U-net with mIoU of 0.7919 vs. 0.8673 for PUTS, ResUnet with mIoU of 0.7914 vs. 0.8673 for PUTS, Unet3+ with mIoU of 0.7998 vs. 0.8673 for PUTS, and DeepLabV3 with mIoU of 0.7932 vs. 0.8673 for PUTS or a mixture of CNN and transformer, as in the case of TransUnet with mIoU of 0.8058 vs. 0.8673 for PUTS, and Swin-Unet with mIoU of 0.8206 vs. 0.8673 for PUTS. Similarly, PUTS outperformed other models that were made of CNNs and transformers on the CVC-ClinicDB dataset, as shown in Table 4.

**Figure 8:**
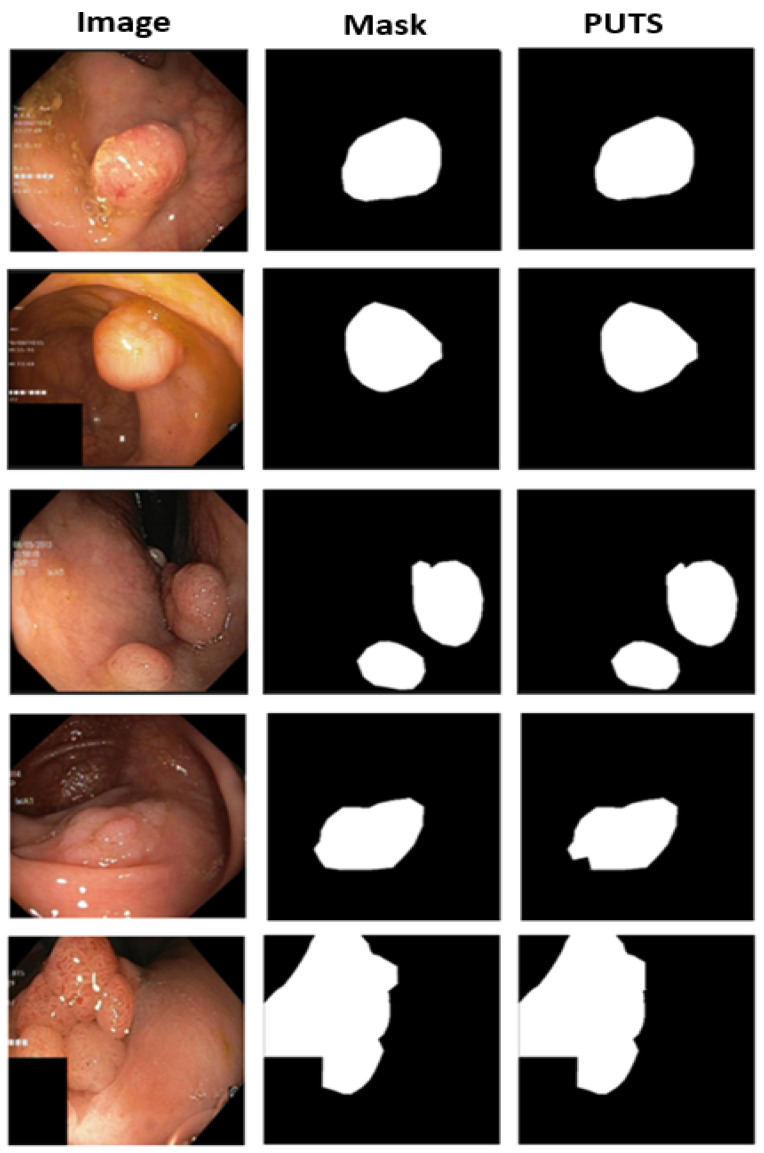
Sample segmentation outputs of PUTS model on Kvasir-SEG dataset.

A visual assessment of the PUTS segmentation output for the Kvasir-SEG dataset is shown in Figure 8. PUTS provides a segmentation output that is almost similar to the segmentation mask, even for challenging images with a sophisticated polyp appearance.

**Figure 9:**
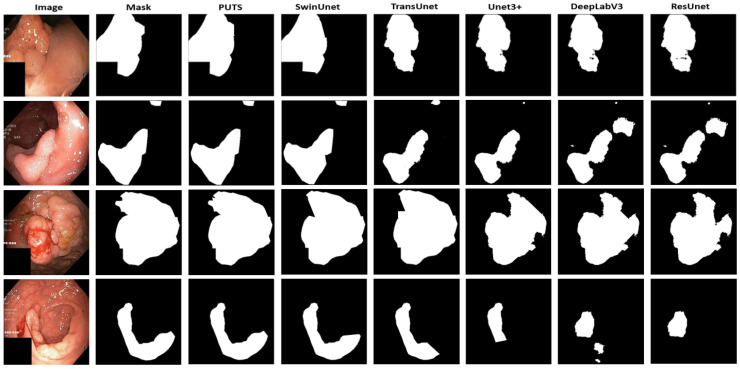
Visual comparison of segmentation output of different models on Kvasir-SEG dataset.

The visual comparison in Figure 9 clearly shows the superiority of PUTS over previous methods for polyp segmentation, providing a segmentation output that is the same as the segmentation mask provided. The polyp image in Figure 9 is a challenging case for the Kvasir-SEG dataset. PUTS captured every detail of the image provided to segment even polyps, which can be difficult for professionals to distinguish.

## 5. Discussion

Both the ViTCol endoscopic pathological findings classification model and PUTS endoscopic polyp segmentation model proposed in this study leveraged the contribution of the STN-based localization module to improve the classification and segmentation performance over other models. The STN offers two advantages. It offers weakly supervised localization without the use of fine-grained supervision, which makes it possible to apply consistency-based regularization, which is particularly advantageous in terms of performance [56]. STN offers weakly supervised localization capabilities, meaning it can identify and localize objects within an image without relying on precise annotations or fine-grained supervision. This capability is particularly advantageous as it allows for the application of consistency-based regularization techniques, which enhance model performance by enforcing consistency across different samples or augmentations of the same data. By leveraging weak supervision and consistency-based regularization, STNs enable more robust and effective learning, especially in scenarios where obtaining precise annotations for training data may be challenging or impractical. This flexibility and performance enhancement contribute to the versatility and effectiveness of STNs in various computer vision tasks. To further investigate if significant performance improvements occur because of the STN, we conducted an experiment using STN and not using STN with the same models and settings for both classification (using three ViT base models) and segmentation (using the Swin-Unet model), as shown in Table 2 and Table 4. As expected, the proposed models with the localization module improved the performance of both classification and segmentation.

Regarding the ViTCol pathological findings classification performance, ViTCol based on vitb_16 outperformed vitb_32 and vitl_32, resulting in reduced computing complexity. The size of the patches utilized in the basic models may be the primary cause of this. The vitb_16 base model utilized a 16 × 16 input patch size, whereas the vitb_32 and vitl_32 base models utilized an input patch size of 32 × 32. The effectiveness and efficiency of the transformer encoder’s attention increase with decreasing patch size. Better results are obtained when extracting local and global characteristics as a result, which consequently improved both classification and semantic segmentation. The network’s size and tendency to overfit the data in comparison to the vitb_16 and vitb_32 models are the other factors, particularly with regard to vitl_32. In addition, the vitb_16 model is better in this situation because it requires less computing power than the vitb_32 and vitl_32 models. For instance, vitb_16 has 12 layers, 768 hidden layer size, 3072 *MLP* size, 12 heads, and 86 million parameters compared to 24 layers, 1024 hidden layer size, 4096 *MLP* size, 16 heads, and 307 million parameters of the vitl_32 model [36].

To further discuss our findings using PUTS, we made a comparison with state-of-the-art polyp segmentation papers published in the past two years on the Kvasir-SEG and CVC-Clinic-DB datasets, as shown in Table 5. To perform the comparison, we followed data settings as in [45], where the datasets were categorized as 90% training and 10% testing. The other settings of the PUTS were set the same as in this study. Following this, the proposed PUTS method, using the Swin-Unet base model, achieved the highest performance on both datasets compared to works published in the past two years. PUTS provided mIoU values of 0.889 and 0.909 for the Kvasir-SEG and CVC-Clinic-DB datasets, respectively. The highest available results published by Li et al. [45] recorded mIoU values of 0.884 and 0.905 for the Kvasir-SEG and CVC-Clinic-DB datasets, respectively. In terms of computational complexity, PUTS showed relatively better results compared to Swin-Unet. PUTS has 36, 142, 374 trainable parameters whereas, Swin-Unet has 35, 072, 374 trainable parameters. For instance, Swin-Unet provided mDic value of 0.8892 on Kvasir-SEG dataset while PUTS provided mDic value of 0.9186. Comparing computational complexity, PUTS provided a 3.306% increment in mDic for a 3.0503% increment in number of training parameters compared to Swin-Unet, showing PUTS’s relative effectiveness in computational complexity.

The proposed ViTCol classification and PUTS segmentation methods can be used in areas other than early diagnosis of endoscopic CRC. In particular, cancer diagnosis imaging modalities in which the tumor is concentrated in a given area could benefit from the method proposed in this study. One such application could be the ultrasound-based early detection of breast cancer. In ultrasound-based early breast cancer detection, the identification of lesions as benign and malignant, and extracting the tumor part from the image, is crucial. The proposed ViTCol and PUTS methods could play a significant role in this task. Moreover, the proposed approach may also be applicable to histopathological image-based cancer diagnosis. In histopathology-based cancer diagnosis, whole-slide images, which are large in size, are used to determine the state of the disease. Studies in this area utilize patches made from whole slide images because the whole slide images possess large pixel sizes that are challenging to process, making them computationally expensive, and the important parts of whole slide images to determine the state of disease can be determined using a portion of the whole slide image. The approach proposed in this study may help tackle this challenge while improving performance.

This study introduced novel deep learning methods, ViTCol, for endoscopic pathological findings classification, and PUTS for endoscopic polyp segmentation, both integrating a Spatial Transformer Network (STN) for localization enhancement. The utilization of STN offers two key advantages: weakly supervised localization and consistency-based regularization. This enables the models to identify and localize objects within images without relying on precise annotations, enhancing performance by enforcing consistency across data samples or augmentations. The findings highlight the versatility and effectiveness of the proposed methods beyond clinical colorectal cancer detection, suggesting potential applications in other cancer diagnosis modalities, such as ultrasound-based breast cancer detection and histopathology-based cancer diagnosis. By effectively localizing tumors and enhancing model performance, the approach holds promise for improving image-based diagnosis processes in clinical settings, aiding healthcare professionals in making informed decisions regarding cancer diagnosis and treatment.

## 6. Conclusions

We designed a novel deep learning method for colorectal cancer early detection using vision transformers. We employed a spatial transformer network for simple localization of tumor prior to the self-attention procedure of vision transformers. We incorporated other gastrointestinal pathological findings that increase the risk of colorectal cancer in our early detection problem in contrast to previous works that only focus on polyp identification. Our classification and segmentation tasks were evaluated on three publicly available endoscopy image datasets. The proposed method achieved superior performance both on the classification and segmentation tasks outperforming state-of-the-art convolutional neural network- and vision transformer-based deep learning approaches in terms of every metric applied. The conventional vision transformer approach often faces lack of localization, and our approach resolved this problem using the spatial transformer network for weakly localizing the tumor area. Our approach can make a significant contribution to image-based diagnosis processes in the clinical setting, assisting professionals in making the best choice regarding colorectal cancer.

## Figures and Tables

**Table 1:** ViTCol performance using different ViT base models.

Model	Accuracy (95%)	AUC (95%)	Sensitivity (95%)	Specificity (95%)	F1 (95%)	MCC (95%)	Kappa (95%)
vitb_16	0.9998 ± 0.001	0.9999 ± 0.001	0.9998 ± 0.001	0.9998 ± 0.002	0.9999 ± 0.001	0.9995 ± 0.002	0.9997 ± 0.001
vitb_32	0.9864 ± 0.002	0.9882 ± 0.001	0.9860 ± 0.002	0.9871 ± 0.003	0.9868 ± 0.002	0.9847 ± 0.003	0.9842 ± 0.002
vitl_32	0.9752 ± 0.001	0.9774 ± 0.002	0.9749 ± 0.001	0.9748 ± 0.002	0.9768 ± 0.002	0.9720 ± 0.002	0.9741 ± 0.002

**Table 2:** Performance of conventional ViT base models.

Model	Accuracy (95%)	AUC (95%)	Sensitivity (95%)	Specificity (95%)	F1 (95%)	MCC (95%)	Kappa (95%)
vitb_16	0.9845 ± 0.001	0.987 ± 0.001	0.9832 ± 0.002	0.9836 ± 0.003	0.984 ± 0.001	0.9817 ± 0.002	0.9826 ± 0.002
vitb_32	0.9684 ± 0.001	0.9742 ± 0.001	0.9644 ± 0.02	0.9642 ± 0.003	0.966 ± 0.001	0.9604 ± 0.003	0.9622 ± 0.002
vitl_32	0.9605 ± 0.001	0.969 ± 0.001	0.9618 ± 0.002	0.9616 ± 0.002	0.9614 ± 0.001	0.9598 ± 0.002	0.9599 ± 0.001

**Table 3:** Performance of CNN-based models.

Model	Accuracy (95%)	AUC (95%)	Sensitivity (95%)	Specificity (95%)	F1 (95%)	MCC (95%)	Kappa (95%)
ResNet50	0.9740 ± 0.001	0.9749 ± 0.001	0.9749 ± 0.002	0.9748 ± 0.002	0.9741 ± 0.001	0.9726 ± 0.003	0.9724 ± 0.002
EfficientNetB2	0.9642 ± 0.005	0.9707 ± 0.004	0.9627 ± 0.003	0.9640 ± 0.004	0.9624 ± 0.006	0.9628 ± 0.003	0.9624 ± 0.003
InceptionV3	0.9702 ± 0.001	0.9718 ± 0.001	0.9694 ± 0.003	0.9697 ± 0.002	0.9702 ± 0.001	0.9659 ± 0.004	0.9648 ± 0.004

**Table 4:** Polyp segmentation performance on Kvasir-SEG and CVC-ClinicDB datasets.

Model	mIoU	mDic	Sensitivity	Specificity	MCC
Kvasir-SEG dataset					
U-net	0.7919	0.7941	0.7908	0.7916	0.7902
ResUnet	0.7914	0.8085	0.7819	0.8021	0.7528
Unet 3+	0.7998	0.8275	0.8142	0.8156	0.8024
DeepLabV3	0.7932	0.8154	0.8068	0.8052	0.8014
TransUnet	0.8058	0.8691	0.8453	0.8512	0.8439
SwinUnet	0.8206	0.8892	0.8734	0.8783	0.8642
PUTS	0.8673	0.9186	0.9095	0.9104	0.9048
CVC-ClinicDB dataset					
U-net	0.8416	0.8892	0.8793	0.8799	0.8724
ResUnet	0.8239	0.8792	0.8674	0.8683	0.8619
Unet 3+	0.8427	0.8904	0.8817	0.8844	0.8786
DeepLabV3	0.8397	0.8829	0.8732	0.8719	0.8694
TransUnet	0.8502	0.8946	0.8915	0.8924	0.8852
SwinUnet	0.8552	0.9053	0.8937	0.8915	0.8906
PUTS	0.9092	0.9484	0.9392	0.9387	0.9317

**Table 5:** Comparison of PUTS with state-of-the-art polyp segmentation works.

Dataset	Paper	Year	mIoU	mDic
Kvasir-SEG	Zhang et al. [57]	2020	0.820	0.884
	Nguyen et al. [58]	2021	0.834	0.894
	Fan et al. [59]	2020	0.840	0.898
	Huang et al. [60]	2021	0.844	0.903
	Wei et al. [61]	2021	0.847	0.904
	Xie et al. [62]	2021	0.864	0.915
	Dong et al. [48]	2021	0.864	0.917
	Li et al. [45]	2022	0.876	0.926
	Liu et al. [63]	2023	0.728	0.803
	Song et al. [64]	2023	0.876	0.924
	Khan et al. [65]	2023	0.874	0.923
	PUTS	2023	0.889	0.935
CVC-ClinicDB	Zhang et al. [57]	2020	0.828	0.883
	Nguyen et al. [58]	2021	0.849	0.904
	Fan et al. [59]	2020	0.849	0.899
	Huang et al. [60]	2021	0.865	0.916
	Wei et al. [61]	2021	0.859	0.916
	Xie et al. [62]	2021	0.882	0.931
	Dong et al. [48]	2021	0.889	0.937
	Li et al. [45]	2022	0.899	0.942
	Liu et al. [63]	2023	0.893	0.941
	Song et al. [64]	2023	0.892	0.940
	Khan et al. [65]	2023	0.903	0.950
	PUTS	2023	0.909	0.949

## Data Availability

In this study, we used a publicly available gastrointestinal endoscopic pathological findings dataset called Kvasir (available online at https://datasets.simula.no/kvasir/ (last accessed on 5 April 2024)) for the classification problem, and two publicly available polyp image datasets, Kvasir-SEG (available online at https://datasets.simula.no/kvasir-seg/ (last accessed on 5 April 2024)) and CVC-ClinicDB (available online at https://www.kaggle.com/datasets/balraj98/cvcclinicdb (last accessed on 5 April 2024)) for the segmentation problem.
